# Drivers of *Echinococcus multilocularis* Transmission in China: Small Mammal Diversity, Landscape or Climate?

**DOI:** 10.1371/journal.pntd.0002045

**Published:** 2013-03-07

**Authors:** Patrick Giraudoux, Francis Raoul, David Pleydell, Tiaoying Li, Xiuming Han, Jiamin Qiu, Yan Xie, Hu Wang, Akira Ito, Philip S. Craig

**Affiliations:** 1 Chrono-environment, University of Franche-Comte (CRNS), Besançon, France; 2 Institut Universitaire de France, Paris, France; 3 Institut National de la Recherche Agronomique (INRA), Unité Mixte de Recherche (UMR), Contrle des Maladies Animales Exotiques et Émergentes (CMAEE), Petit-Bourg, Guadeloupe, France; 4 Sichuan Center for Disease Control, Chengdu, China; 5 Qinghai Center for Disease Control, Xining, China; 6 Institute of Zoology, Chinese Academy of Sciences, Beijing, China; 7 Department of Parasitology, Asahikawa Medical University, Asahikawa, Japan; 8 School of Environment and Life Sciences, University of Salford, Salford, United Kingdom; Jiangsu Institute of Parasitic Diseases, China

## Abstract

**Background:**

Human alveolar echinococcocosis (AE) is a highly pathogenic zoonotic disease caused by the larval stage of the cestode *E. multilocularis*. Its life-cycle includes more than 40 species of small mammal intermediate hosts. Therefore, host biodiversity losses could be expected to alter transmission. Climate may also have possible impacts on *E. multilocularis* egg survival. We examined the distribution of human AE across two spatial scales, (i) for continental China and (ii) over the eastern edge of the Tibetan plateau. We tested the hypotheses that human disease distribution can be explained by either the biodiversity of small mammal intermediate host species, or by environmental factors such as climate or landscape characteristics.

**Methodology/findings:**

The distributions of 274 small mammal species were mapped to 967 point locations on a grid covering continental China. Land cover, elevation, monthly rainfall and temperature were mapped using remotely sensed imagery and compared to the distribution of human AE disease at continental scale and over the eastern Tibetan plateau. Infection status of 17,589 people screened by abdominal ultrasound in 2002–2008 in 94 villages of Tibetan areas of western Sichuan and Qinghai provinces was analyzed using generalized additive mixed models and related to epidemiological and environmental covariates. We found that human AE was not directly correlated with small mammal reservoir host species richness, but rather was spatially correlated with landscape features and climate which could confirm and predict human disease hotspots over a 200,000 km^2^ region.

**Conclusions/Significance:**

*E. multilocularis* transmission and resultant human disease risk was better predicted from landscape features that could support increases of small mammal host species prone to population outbreaks, rather than host species richness. We anticipate that our study may be a starting point for further research wherein landscape management could be used to predict human disease risk and for controlling this zoonotic helminthic.

## Author Summary

The loss or gain of certain host species may either dilute or amplify the risk of pathogen and parasite infection through direct or indirect effects. The relative contribution of host communities combined with climate and landscape characteristics on non-vector-borne parasite transmission to humans has been a relatively neglected area of investigation. Here we show that zoonotic transmission of the cestode *E. multilocularis* in China was not directly correlated with small mammal host species richness, but spatially correlated with alpine meadows, forest characteristics and rainfall that confirmed hotspots of human disease in a 200,000 km^2^ region of the eastern Tibetan plateau. Our findings indicate more intensive transmission in homogeneous landscapes with larger areas of optimal habitats for one or some host species in low diversity small mammal communities, making multi-annual population outbreaks more likely. Landscape features that could support large population outbreaks of small mammal host species were better predictors of *E. multilocularis* transmission to humans than indices of host species richness per se. Our results support the notion that landscape, small mammal host biodiversity and their population dynamics may protect humans from zoonotic parasite transmission where they prevent population outbreaks of a few specific small mammal host species.

## Introduction

Ecologic systems are nested within one another. This well-known fundamental hierarchical organization [1] is easy to detect in nature but has been generally undervalued as a source of influence on the structure and development of pathogen transmission patterns, and also as a means of understanding the crucial connections between local processes and large-scale distribution patterns. At a community level, Guernier et al. [2] explored the worldwide distribution of human parasitic and infectious diseases (PID) and found that, after correcting for cofactors, PID richness (as for free-living species), was strongly correlated with latitude: PID species diversity decreased as one moved from the equator, and the strongly nested pattern of their global distribution was confirmed. They also pointed out how, along such gradient, the maximum range of precipitation and monthly temperature might be intimately connected in generating the observed pattern of disease diversity. This similarity in the diversity patterns of free living organisms and PIDs suggests that common processes are at work which might be explained at large by the climatically based energy hypotheses (energy availability generates and maintains species richness gradients) [2]. However, for a given PID species distribution patterns at regional scales may be more complex. Transmission may depend not only on species richness but also on host assemblage composition [3]. For instance, reducing host diversity can increase disease transmission when the lost species are either not hosts or suboptimal for the pathogen, especially when population size of optimal hosts are inversely correlated to species richness. On the other hand, a large number of competent host species may provide a much more stable transmission system for transmission, that is robust against environmental disturbances, anthropogenic or natural, that temporarily decrease the density of some host populations. However, empirical examples of the relationships between host biodiversity and parasite transmission are still relatively rare, in part because suitable datasets that may allow comparisons are deficient [4].

Further complexities are encountered when the distribution range of a pathogen covers a large number of host communities and climatic zones. Such a wide-extent distribution-range consists of a nested hierarchy of transmission systems that can be inter-connected in space and time via dispersion [5]. At these scales, datasets with high resolution and precision, describing hosts, disease distribution and environmental factors (such as climate and land cover) are most often heterogeneous.

**Figure 1:**
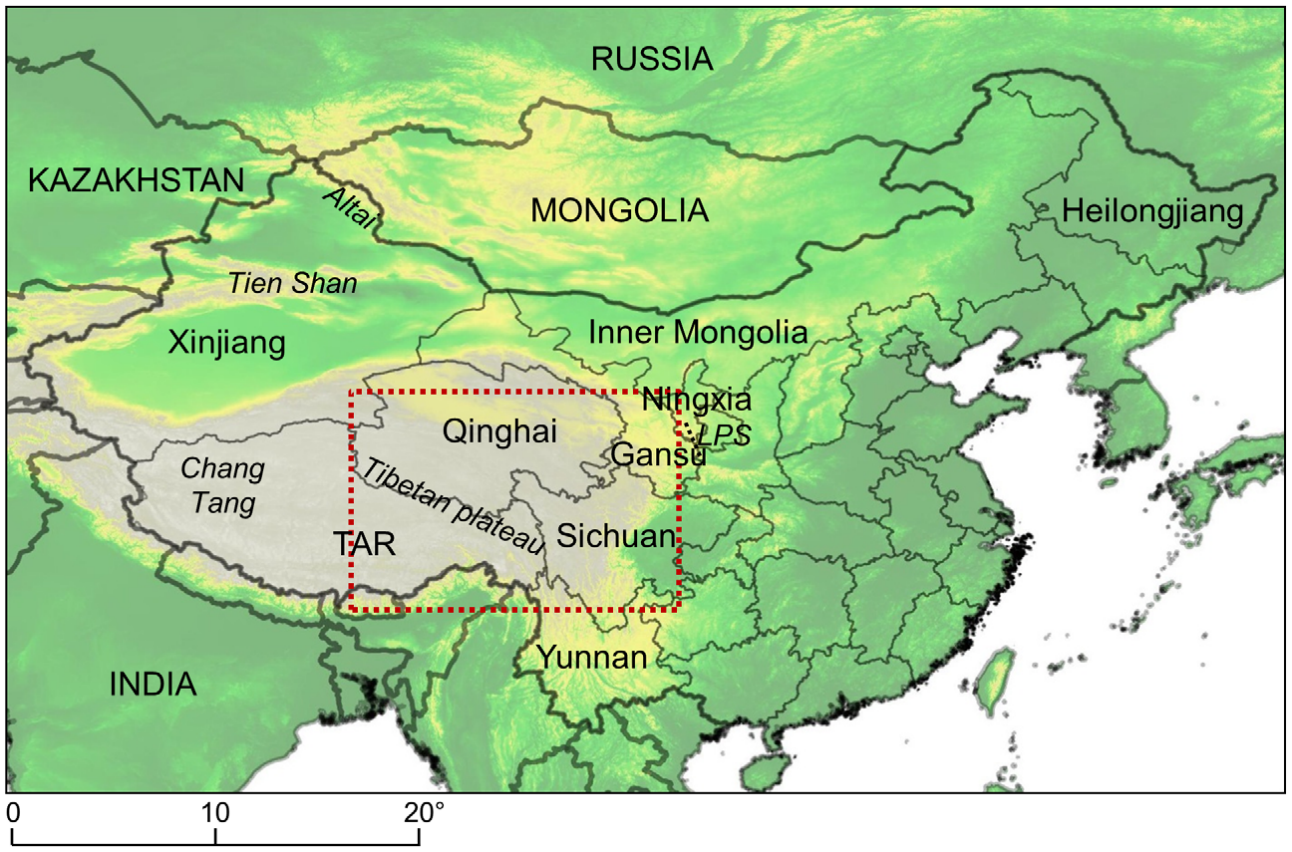
Map of China and main locations cited in the text. Dotted line, area where regional predictions were computed (see Figure 7), TAR, Tibetan Autonomous Region, LPS, Liupan Shan (Liupan Mountains). Coordinates reference system in degrees (WGS84). The background is Global Land One-kilometer Base Elevation model, provided by the US National Oceanic and Atmospheric Administration.

Human alveolar echinococcocosis (AE) is a highly pathogenic parasitic disease caused by the larval stage of the cestode *Echinococcus. multilocularis*, which usually results in a slow-growing multivesicualted tumor-like lesion in the liver of cases. The parasite's life-cycle can exploit a large number of small mammal intermediate hosts (>40 species known to date) and several definitive host species (e.g. foxes, coyotes, wolf, dog, etc.). Human infection arises from accidental ingestion of *E. multilocularis* eggs from direct contact, or via food contaminated by carnivore definitive host faeces. Although patchy, its distribution range covers the Northern hemisphere from the Arctic to the 28^th^ parallel on the Tibetan plateau. In Eurasia, although other carnivores can theoretically sustain the transmission of *E. multilocularis*, the whole range of the parasite is actually included in the range of its main definitive host, the red fox, *Vulpes vulpes*, except on the Tibetan plateau where the cestode circulates through the Tibetan fox, *Vulpes ferrilata* [6]. In contrast, throughout Eurasia, *E. multilocularis* transmission is sustained by a large variety of different small mammal communities [7]. Within Eurasia, continental China stretches from Siberia in North Xinjiang, Inner Mongolia and Heilongjiang to tropical rain forest in Yunnan, and includes high altitude areas such as the Tibetan plateau, several deserts (some of them below sea level), coastal and agricultural areas (Figure 1). Based on mammal and plant species comparisons a total of 25 biogeographical regions and 77 sub-regions are defined [8]. Transmission of *E. multilocularis* is sustained in diverse small mammal communities of western and northern China. While some of these communities have been investigated [5], [9] many remain unexplored.

Here we examine the distribution of human AE disease in China on two spatial scales (continental and regional) and test the hypotheses that human disease distribution can be explained by the distribution of intermediate host species richness (i.e. host biodiversity) or by environmental factors such as climate and land cover or an interaction between them. Our central hypothesis is that human AE distribution is best explained by considering the impact of landscape on low species-rich communities prone to population outbreaks, rather than host diversity per se. In that case, for endemic areas, basic information on land cover and climate could be used as a proxy to predict high risk transmission systems for this pathogenic helminthic zoonosis.

## Materials and Methods

### Continental China

#### Small mammal data

Small mammal distributions across China were obtained from a digital version of maps published by Smith et al. [10]. Species nomenclature were standardized according to Wilson and Reeder [11]. Since the main intermediate hosts of *E. multilocularis* are small mammals, the following taxa were selected: Order Rodentia (family Sciuridae -excluding flying squirrel-, Gliridae, Castoridae, Dipodidae, Platacanthomyidae, Spalacidae, Cricetidae and Muridae), Order Lagomorpha (family Ochotonidae and Leporidae), Erinaceomorpha (family Erinaceidae), 0rder Soricomorpha (family Soricidae and Talpidae), totaling 274 species. A total of 967 point locations were defined on a regular grid (100 km mesh, coordinates reference system: UTM47) covering continental China and a buffer of 100 km radius centered on each node. The ecology and population dynamics of small mammal species is still little known in China. However, some species whose population outbreaks impact agriculture or forestry have been identified as pest species in the literature [5], [10], [12]–[15]. Evidence of interactions between land cover, human activities and small mammal population dynamics has been provided in Europe and Japan for a number of grassland and forest species [16]–[18]. Therefore, in the present study, the category ‘pest species’ was considered as a surrogate for those small mammal species whose populations are prone to outbreak and reach large densities locally. Intermediate host species were those previously reported for *E. multilocularis* [5], [7], [19]–[22]. Thus the number of species (species richness) belonging to the following categories were computed within each buffer: ‘all species’, ‘not flying small mammals’, ‘species known to be intermediate hosts’, ‘species known to be pests’, ‘species known to be intermediate hosts and pests’.

#### Environmental and climate data

Monthly and yearly average rainfall and temperature for China were extracted from the 0.5 degree [23] gridded dataset of the FAO Global maps (http://www.fao.org/geonetwork). Altitude maps were derived from the Global Land One-km Base Elevation (GLOBE) Project [24]. Using the same grid and buffers as above we computed the mean, median, minimum and maximum altitude. Information on land cover was obtained from the Global Land Cover 2000 project [25]. It was based on Spot Vegetation data in the period 1.1 to 31.12.2000 and consisted in 24 land-cover classes mapped at a pixel resolution of 1 km and was designed to cover the full diversity of land-cover found across China, using the FAO Land Cover Classification System.

#### Human data

Zhou et al. [26], made an extensive literature search that identified counties (administrative subdivisions of provinces in China) where one or more human AE cases were recorded and derived a distribution map. This map, following Danson et al. [27] was used in the present study.

### Eastern Tibetan plateau

#### Human data

Mass screening programs for human abdominal echinococcosis were performed from 2002 to 2008 using portable ultrasound in communities that comprised a total of 94 villages, located in Western Sichuan and Qinghai provinces. Persons with confirmative or suspected AE or cystic echinococcosis (CE, caused by *E. granulosus*) lesions or with other space-occupying lesions in the liver were asked to give a five ml venous blood sample for detection of *Echinococcus* antibodies using ELISA with recombinant AgB as antigen for CE or AE, and ELISA with recombinant Em18 antigen for AE [28]. In cooperation with County level Centers for Disease Control (CDC) and local health administrators, information about the purpose of the screening program was spread to the villagers. People screened were self-selected volunteers and agreed by informed consent. For each participant a questionnaire was completed with the help of Tibetan registration auxiliaries, to obtain information on gender, age, occupation and ethnicity (see [28] for further details).

#### Ethics statement

Approval for the surveys was given by the relevant ethics Committee of the Centers of Disease Control in Sichuan and Qinghai, China. Written consent was obtained from all adult participants and from parents of minors who agreed to participate [28]. Persons with confirmative or suspected AE or CE were assured free diagnosis and medical treatment with long-term albendazole drug therapy for echinococcosis if diagnosis was positive [28]. Recommendation was also provided for possible surgical intervention (cyst/lesion removal or drainage) if appropriate. Persons with other infections or medical conditions were examined and referred to local health clinics for further investigation or treatment.

#### Data handling and models

Land cover was estimated as the proportion of pixels belonging to a Global Land Cover 2000 category in question within a circular buffer centered at a given pixel. Elevation, yearly rainfall and yearly average temperature, were extracted from a 0.1 degree gridded dataset of the FAO Global maps, and assigned the median pixel values within the buffer. Such metrics vary with respect not only to landscape composition but also to the buffer radius R. Therefore radii of 10 km, 20 km, 50 km and 100 km were considered. This was repeated for every pixel in a 50×32 grid with a 20×20 km resolution. To avoid resolution differences between human data collected in each village and environmental data available for every pixel of the 20×20 km resolution grid, human data were assigned to the nearest pixel centroid. Generalized additive mixed models (GAMMs) with a logistic link function [29] were used to investigate non-linearities between age, environmental variables and AE status (presence/absence of hepatic AE) of subjects. A within-pixel (or within-village for age) random effect was added to account for pixel or village specific peculiarities in prevalence arising from unobserved pixel or village specific factors. Land cover effects (within buffer percentage of alpine meadows, alpine steppe – termed ‘alpine and sub-alpine plain grass’ in the global land cover 2000 nomenclature –, forest), altitude and rainfall, were modeled similarly for all buffer sizes. The ratio of farmland was nominal within this range of altitude and was not kept for this analysis. The most pertinent value for buffer radius R was identified via Akaike Information Criterion (AIC) based model selection. Selected GAMMs were further subjected to Bayesian analysis using the software BayesX [30], [31] which enabled the inclusion of a spatial random effect to account for otherwise unexplained between-pixel spatial autocorrelation. The spatial random effect was modeled as a Markov random field [32], [33] on the 20 km resolution grid. Non-linearity was modeled using a degree 3 P-splines on 20 equidistant knots and a second order random walk was used to penalize against over fitting, [34], [35]. Non-informative inverse gamma priors were assumed for variance components of the P-spline and random effects with shape and scale hyper-parameters initialized at 0.001). The parameter space of the Bayesian GAMMs was sampled using Markov Chain Monte Carlo (MCMC) techniques. For each model, MCMC was run for 500,000 iterations, discarding an initial burn-in period of 50,000 iterations and sub-sampling every 400th sample thereafter. Model comparison was performed using the deviance information criterion (DIC) [36] with model selection based upon the model returning the lowest DIC. Coefficients and corresponding credibility intervals of selected models were examined. The probability for each linear coefficient to be either strictly positive or strictly negative was computed from their empirical density distribution, considering that the direction of the effect could be expected based on earlier works and preliminary statistics.

Computing and graphical displays were performed using BayesX 2.0.1, R 2.11.1 [37], the R packages BayesX 0.2–4 [38], coda [39], fields 6.3 [40], gstat 0.9–69 [41], mgcv 1.6–2 [29], maptools 0.7–34 [42], rgdal 0.6–2 [43], sp 0.9–65 [44], splancs 2.01–27 [45] and pgirmess 1.4.7 [46].

## Results

### Continental China

**Figure 2:**
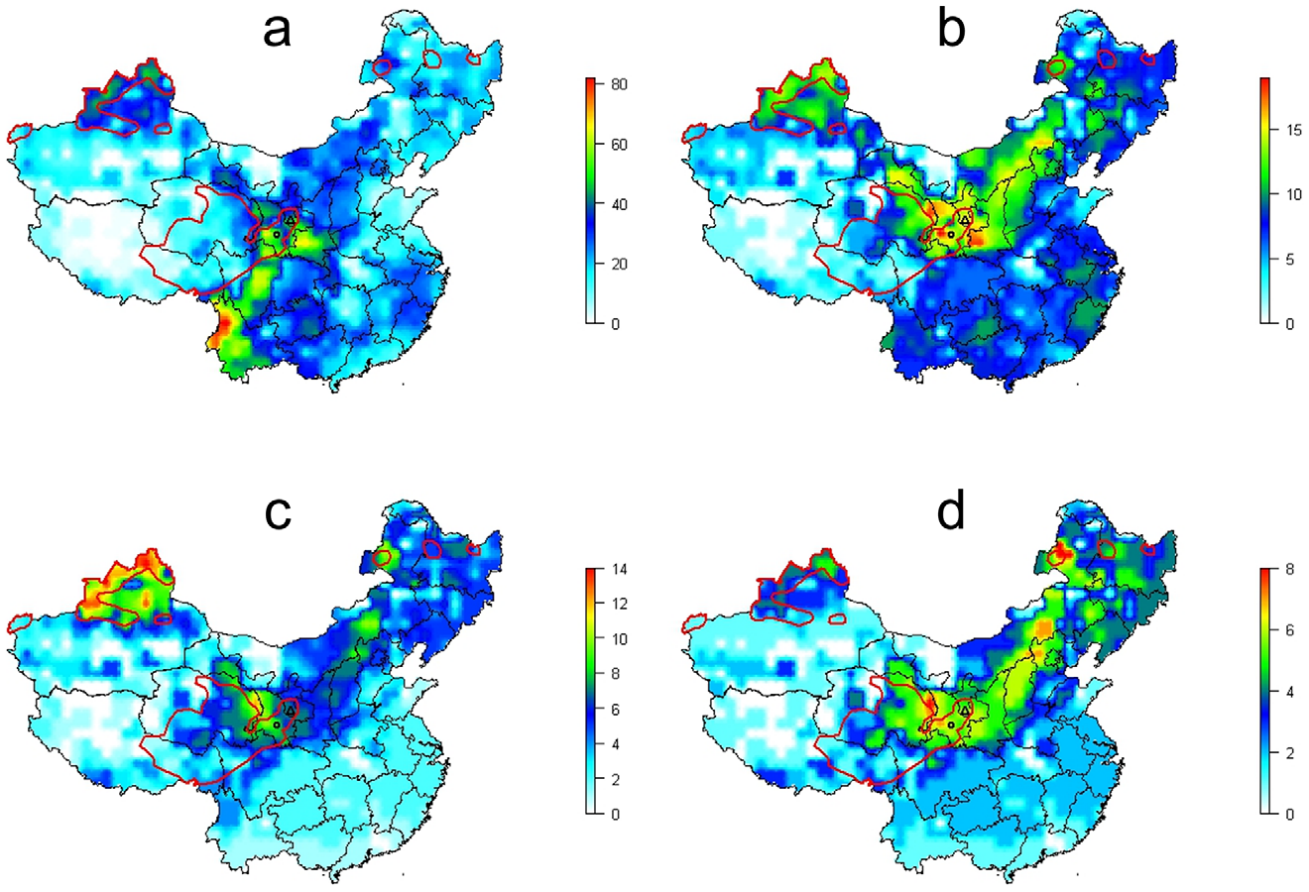
Biodiversity of non-flying small mammals. a, all species; b, pest species; c, intermediate hosts species; d, pest and intermediate host species; red line, limits of human AE areas; circle, Zhang-Puma counties, Gansu; triangle, Xiji-Guyan counties, Ningxia. Vertical bars represent the number of species.

**Figure 3:**
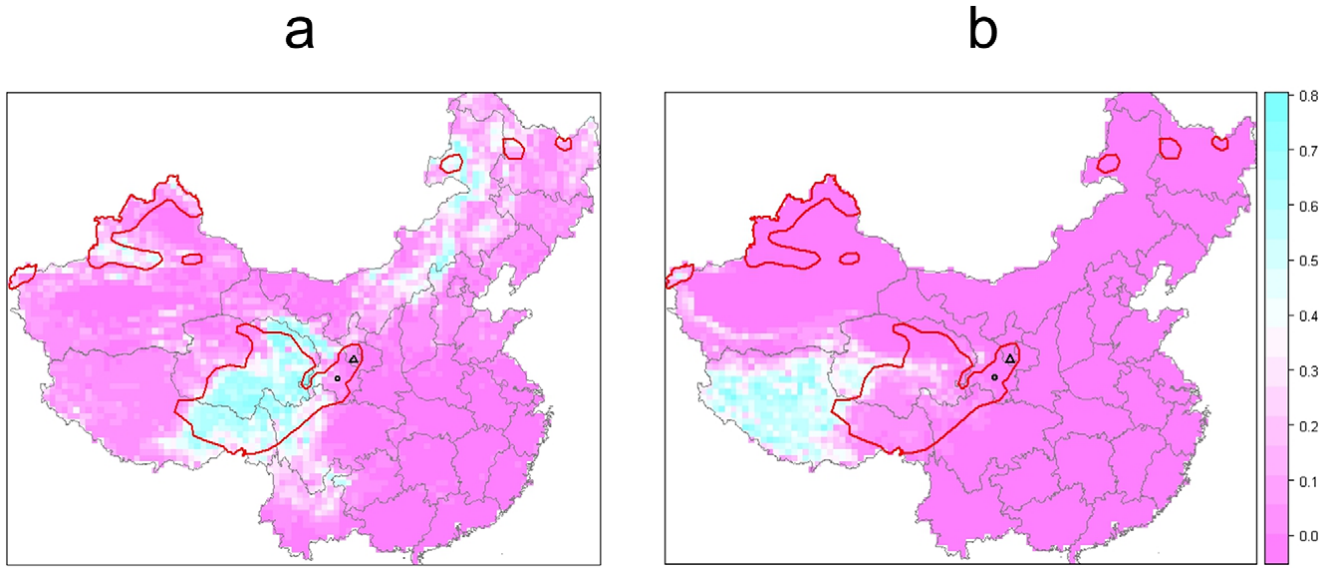
Grasslands in China (ratio of total land in a 100 km radius buffer). a, ‘meadows’ and ‘subalpine meadow’; b, ‘alpine and sub-alpine plain grass’ (Global land cover class terminology) actually corresponding to high altitude steppes; red line, limits of human AE areas; circle, Zhang-Puma counties, Gansu; triangle, Xiji-Guyuan counties, Ningxia.

Continental maps of China for distribution of rainfall, altitude and temperature can be found in Supplementary Material. In summary these abiotics factors did not correlate well to human AE disease distribution. Furthermore, the continental distribution of human AE was not clearly correlated to small mammal species richness, even considering the distribution and number of species that are known to be potential intermediate hosts for *E. multilocularis* or pest species (Figure 2). However, the overlap between the spatial distribution of human AE and the spatial distribution of a combination of two Global Land Cover 2000 categories, i.e. ‘meadows’ and ‘alpine and subalpine meadow’ was very clear. The main endemic areas correspond to the central and eastern Tibetan meadows, those of the Tien Shan and Altai Mountains and northern Inner Mongolia (Figure 3a). On the Tibetan plateau, the human AE endemic foci corresponds to the Global land cover category ‘alpine and subalpine meadow’, characterized by alpine meadows densely covered with thick perennial sedges (*Kobresia* spp) and various forbs, lying generally below 4500 m as described by Schaller [47]. In the western part of the human AE focus, alpine meadow becomes largely riparian with streams, seepages, swamps and lakes. The area is characteristically composed of a 10–40 cm thick moisture retaining sod layer providing a long growing season. Livestock tend to concentrate on grassy habitat (28–70 animals/km^2^) and continual grazing and trampling coupled with solifluxion, cause extensive erosion. This anthropogenic activity helps to maintain extensive open habitats favorable to the population surges of the plateau pika *(Ochotona curzoniae)*, a known intermediate hosts of *E. multilocularis* [48]. By contrast, human AE distribution did not correlate with the Global Land Cover 2000 category ‘alpine and sub-alpine plain grass’ (Figure 3b). This category, lying generally between 4500 and 5000 m, corresponds to an Alpine steppe, dry, cold and windy. Plant coverage is sparse (*Stipa spp, Festuca spp, Poa spp, Carex moorcroftii*) seldom more than 30%, and soil is poor without a sod layer [47].

Despite its great size (several 100,000 km^2^), the Eastern Tibetan plateau focus of human AE disease correlates well with Alpine meadows. The apparent absence of human AE cases on the western and north western Tibetan plateau may simply be due to the fact that human population density decreases from east to west to low population areas of high altitude desert (i.e. Chang Tang area). From east to west on the Tibetan plateau, climatic conditions get drier and colder, which impacts grass growth and yak/goat/sheep survival on which human populations depend. This aridity can also be expected to affect *E. multilocularis* egg survival. On the other hand, AE cases may possibly just be undetected in small discrete nomadic Tibetan populations of the western Tibetan plateau, since they are typically isolated from public health facilities and no mass screening has occurred to date west of Naqu in Tibet Autonomous Region. We return to this issue in the following section.

Based on this China-wide continental scale analysis, some interesting contrasting results were apparent. Regional areas of south Gansu province (i.e. Zhang-Puma counties), and south Ningxia region (i.e. Xiji-Guyuan counties, close to the North Liu Pan Mountains) were not expected to have large number of human AE cases. However, local foci over an area of 400–2500 km^2^ were confirmed to have significant AE prevalence in humans reaching an average of 4.1% and 3.0% respectively. These largely montane agricultural zones are considered to be significantly influenced by anthropogenic landscape disturbance caused by deforestation [9], [49], [50]. This process led to the regional opening of forest areas to agriculture, with transitional stages of grassland and pastures triggering small mammal population outbreaks, thus potentially fostering transmission of *E. multilocularis* locally over a relatively short time-span (10–20 years) when optimal small mammal habitats become temporarily available. Thus, in both the provincial Gansu and Ningxia endemic zones, risk of human AE prevalence increased during the 1970s–90s in response to anthropogenic local landscape changes. However these landscape changes cannot be seen on the coarse resolution land use map.

### Eastern Tibetan plateau

**Figure 4:**
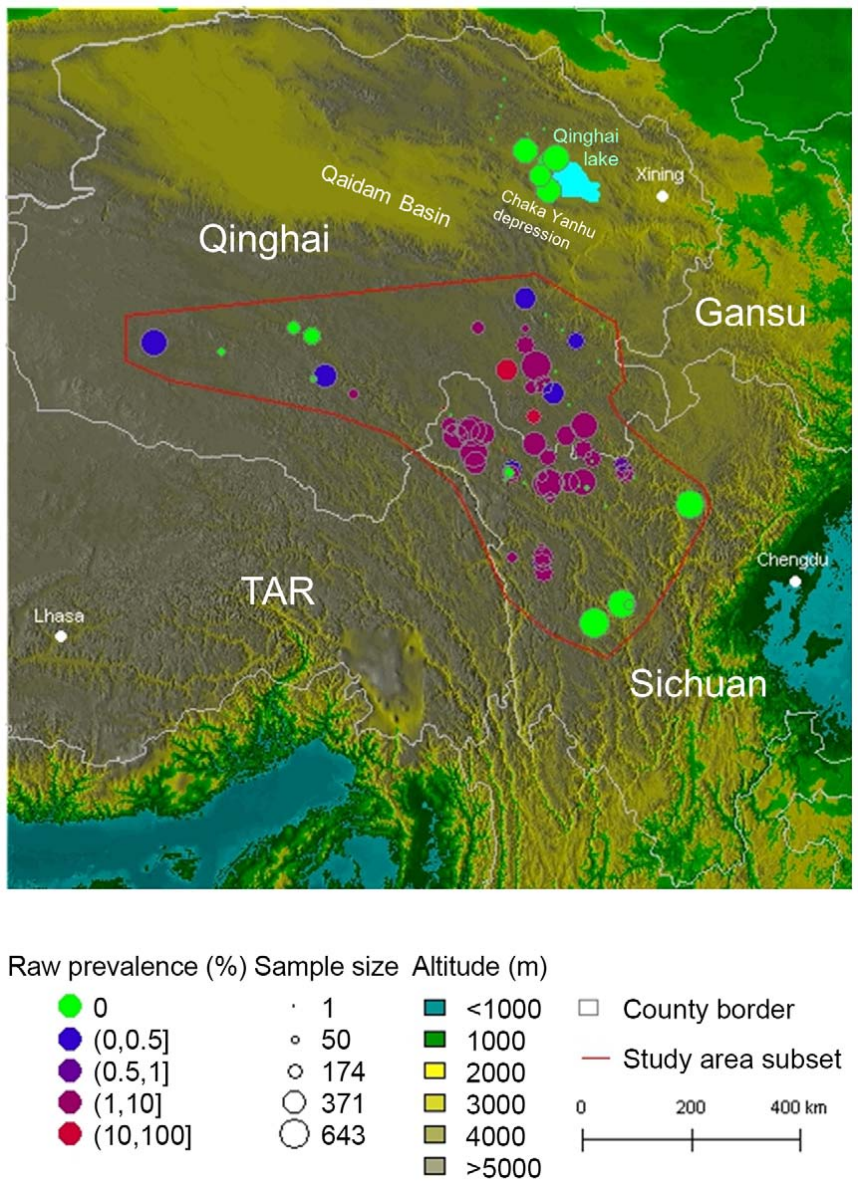
Raw prevalence and sample size of the 94 villages.

**Figure 5:**
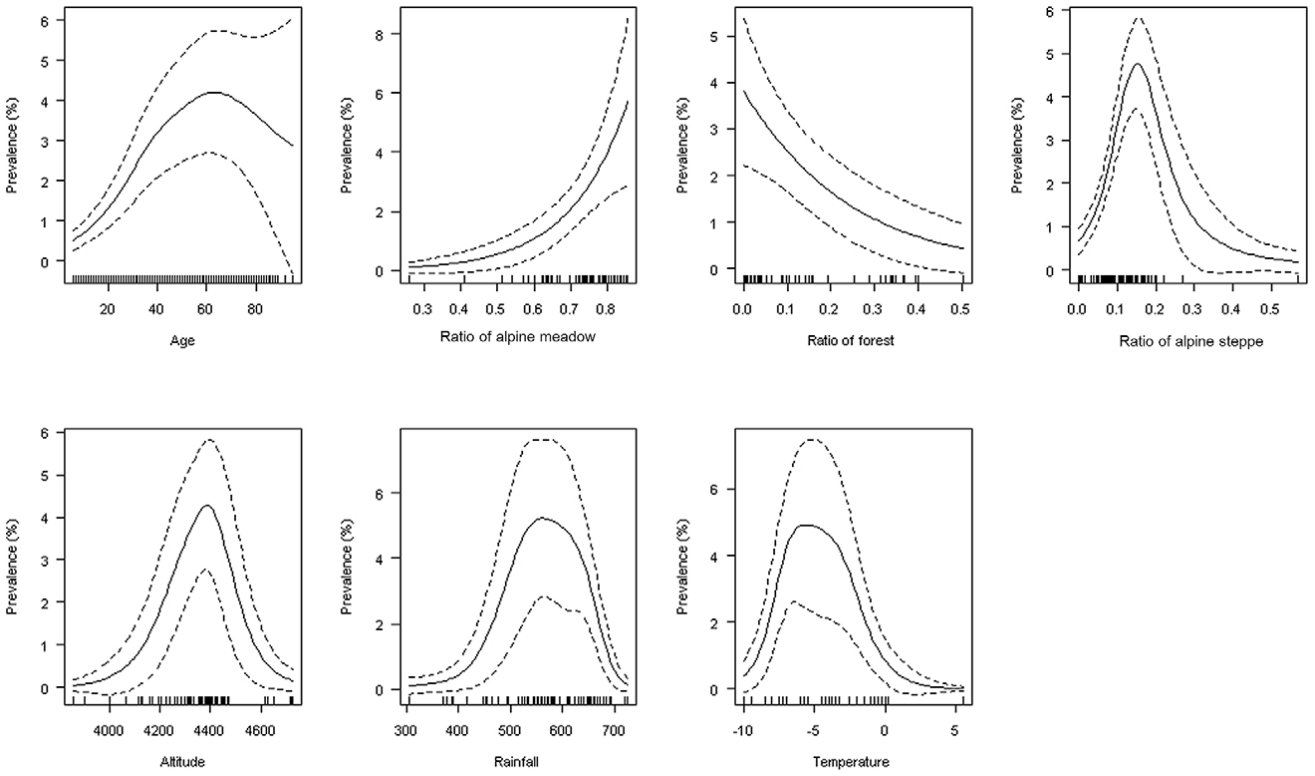
Relationships between age, environmental factors and AE status. Predicted values (solid line) and 95% CI (dotted line) were computed using a general additive mixed model with a binomial link, a cubic regression spline smoothed term on age and a random effect on villages [29]. The rug on the abscissa represents the observations (some at the same position may overlap).

A total of 17,589 people were screened for hepatic echinococcosis in 94 villages and the overall prevalence of alveolar echinococcosis was 3.28% (3.02–3.56, 95%CI). Despite substantial sample sizes, no cases were detected to the west of Qinghai Lake (Figure 4). People screened in this area (n = 1,975) were 95.5% Tibetan (71.7% herdsmen, 25.6% children). Geographically isolated from the southern hotspot by the lower Qaidam Basin and the Chaka Yanhu depression, this area was not considered for further analysis (see study area subset Figure 4). Thus, the resulting filtered data set included 15,614 people and 81 villages, with a raw prevalence of 3.7% (3.41–4.01, 95%CI) (Table 1). Univariate analysis detected significantly higher AE prevalence among females than among males (X^2^ = 11.2, df = 1, p = 0.0008) and among Tibetan than among Han ethnic groups (X^2^ = 6.05, df = 1, p = 0.01). Evidence of occupation differences was found (X^2^ = 129, df = 5, p<0.000001), with AE prevalence greater in herdsmen compared to a pool of other categories (X^2^ = 29.5, df = 1, p<0.000001) and lower in employee (X^2^ = 5.57, df = 1, p = 0.02), student and children (X^2^ = 41.9, df = 1, p<0.000001), semi-herdsman (X^2^ = 7.02, df = 1, p = 0.008) and farmers (X^2^ = 32.1, df = 1, p<0.000001). Table 2 shows how different occupations were represented between ethnic groups. The link between age categories and some occupations (e.g. ‘student and children’) is obvious. A non-linear relationship of human AE prevalence to age was found (Figure 5).

**Table 1:** Prevalence for the current subset excluding Qinghai Lake area (see text).

Subset	c/n	Prev%	Subset	c/n	Prev%	Subset	c/n	Prev%
Full data	577/15614	3.70						
		(3.41, 4.01)						
Male	250/7839	3.19	Female	327/7764	4.21			
		(2.82, 3.61)			(3.78, 4.69)			
Han	9/545	1.65	Tibetan	568/15069	3.77			
		(0.8, 3.23)			(3.47, 4.09)			
Farmer	8/1190	0.07	Semi-herdsmen	8/539	1.48	Student and children	62/3397	1.82
		(0.03, 1.38)			(0.69, 3.02)			(1.41, 2.35)
Employee	27/1136	2.38	Other	56/1363	4.10	Herd people	415/7978	5.20
		(1.60, 3.49)			(3.15, 5.34)			(4.73, 5.72)

c/n indicates the number of AE cases in a subsample of size n; 95 percent confidence interval between parentheses.

**Table 2:** Percentage of occupations in each ethnicity category.

	Han	Tibetan
employee	57.5 (313)	5.5 (823)
farmer	5.3 (29)	7.7 (1161)
herdsman	1.7 (9)	52.9 (7969)
other	29.2 (159)	8 (1204)
semi-herdsman	1.7 (9)	3.5 (530)
student and children	4.6 (25)	22.4 (3372)

Size of the subsample between parentheses; the current subset excludes Qinghai Lake area; columns sum to 100%.

The buffer radius maximizing the likelihood of the GAMM was found to be 100 km for all land cover variables, altitude, rainfall and temperature. Figure 5 shows the relationships between AE prevalence in humans and environmental variables. AE prevalence was found to increase exponentially with the ratio of Alpine meadows and to decrease with the ratio of forest (corresponding to a linear relationship on the linear predictor – data not shown). Human AE prevalence increased with the percentage of Alpine steppe to a maximum at 15.6% of total land cover, and then decreased to low prevalence. Similar patterns were obtained for rainfall, altitude, and temperature with maximum at 564 mm, 4385 m and −5.62°C respectively. This suggests an increased risk for human AE in areas with a larger percentage of alpine grassland at altitudes ranging between 4200–4600 m. In comparison, landscapes dominated by alpine steppe at higher altitude, mostly observed in the western part of the study area, appeared to be clearly sub-optimal.

We included in models either altitude as a proxy for other ecological parameters (such as rainfall, temperature and other unknown factors) or rainfall and temperature as potential ecological factors impacting directly e.g. *Echinococcus* egg survival. Models containing non-linear effects on all three variables frequently gave rise to computational difficulties and are not reported here. Models including the spatial random effect consistently returned lower DICs than their non-spatial counterpart (see Table S1 in Supplementary material for the list of models). Evidence for the importance of a non-spatial (pixel) random effect was less overwhelming. DIC comparisons show that 5 models among the 33 fitted were quasi-equivalent with a DIC difference lower than 2. All of them included all variables related to human populations (ethnicity, gender, age, occupation), all landscape variables and at least one variable related to climate or altitude. The mean and 95% credibility intervals of posterior samples for regression coefficients, and the probability that the coefficient be ≤0 (Tibetan, female, herders, meadows) or ≥0 (forest), are shown in Table 3. The proportion of positive coefficient samples were > = 0.98 for Tibetan, > = 0.99 for female and < = 0.03 for forest in all models. 95%CIs for meadow coefficients did not exclude zero or negative values when forest was included, however, >95% of posterior samples for that coefficient were positive. Although the coefficient for herdsmen was greater than zero in less than 90% of samples, dropping this term from models largely increased DIC.

**Table 3:** Summary statistics of posterior samples for regression coefficients of the 5 best models.

Model		Intercept	Tibetan	Female	Herdpeople	Ratio meadows	Ratio Forest
1	Mean	1.18	0.77	0.28	0.12	−8.72	−10.50
	CI 95%	(−6.36, 8.32)	(0.17, 1.52)	(0.11, 0.45)	(−0.12, 0.36)	(−18.95, 1.65)	(−18.51, −2.47)
	p	0.380	0.005	<0.0001	0.159	0.952	0.004
2	Mean	1.51	0.76	0.28	0.12	−9.20	−10.52
	CI 95%	(−5.99, 9.57)	(0.18, 1.47)	(0.11, 0.45)	(−0.11, 0.36)	(−20.73, 1.06)	(−18.11, −2.92)
	p	0.342	0.003	<0.0001	0.155	0.965	0.007
3	Mean	1.30	0.76	0.28	0.12	−10.28	−10.03
	CI 95%	(−6.75, 9.2)	(0.12, 1.52)	(0.1, 0.46)	(−0.12, 0.35)	(−21.03, 0.82)	(−20.83, 0.08)
	p	0.378	0.013	0.004	0.144	0.966	0.026
4	Mean	0.68	0.78	0.28	0.11	−8.72	−9.40
	CI 95%	(−6.7, 8.08)	(0.1, 1.57)	(0.11, 0.44)	(−0.14, 0.35)	(−18.42, 1.11)	(−18.41, −0.79)
	p	0.422	0.011	<0.0001	0.180	0.953	0.020
5	Mean	0.96	0.78	0.28	0.12	−9.79	−9.17
	CI 95%	(−6.38, 9.02)	(0.1, 1.49)	(0.11, 0.44)	(−0.12, 0.37)	(−20.99, 0.23)	(−19.17, 0.45)
	p	0.413	0.012	0.001	0.143	0.971	0.029

p, the probability that the coefficient be ≤0 (Tibetan, female, herd people, meadows) or ≥0 (forest); CI 95%, 95 percent credibility interval.

**Figure 6:**
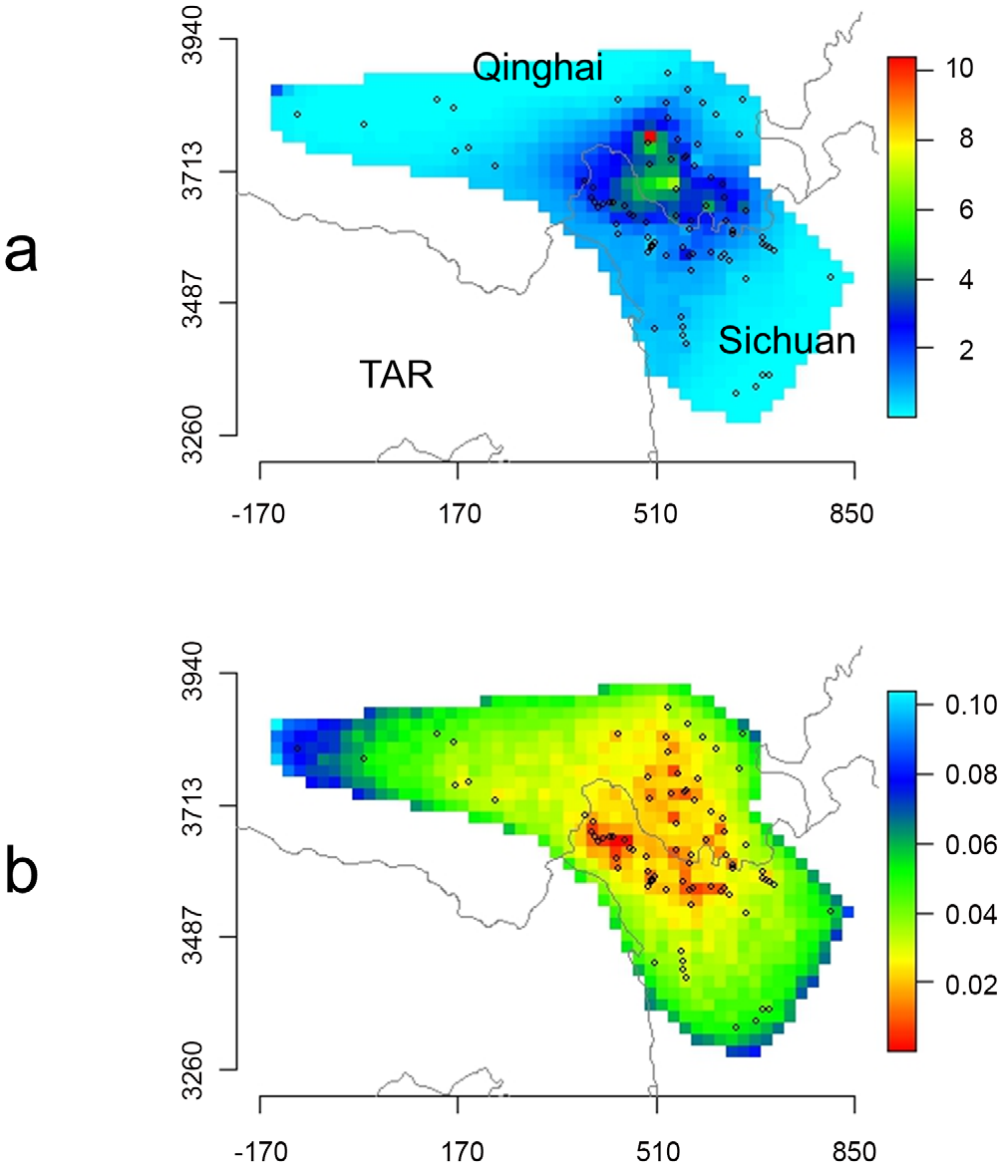
Predictions of human AE prevalence. Bounding box is visible in Figure 1. (a) prevalence, (b) range of the credibility interval from the posterior simulation of the spatial random effect (on the linear predictor scale). Units of horizontal and vertical axes are 1/1000 UTM tile 47, the area corresponds to the area subset of Figure 4. Trend terms were those of model 5. The model succeeds in smoothing the raw prevalence to within a plausible range. The main hotspot is identified in the area at the south-east border of Qinghai and at the north-west of Sichuan. Axes units are in kilometers (UTM tile 47).

For prediction of human AE disease risk, model 5 was selected in order to avoid altitude (a proxy variable whose importance may change with latitude outside of the study area), and in order to maintain the ecologically meaningful variables such as rainfall and temperature which appear not to lead to excessive over-parameterization, even keeping a pixel random effect. Posterior means and standard deviations of the variance parameters for the P-splines and Markov random field were 0.0147 (sd 0.02306) for age, 0.3831 (sd 0.6105) for rainfall, 0.1468 (sd 0.5690) for temperature, 1.2036 (sd 0.5164) for pixel spatial random effect, and 0.0586 (sd 0.0963) for pixel random effect. Figure 6a presents the predicted prevalence for a hypothetical 31.6 year old male, non-Tibetan, non-herdsman (corresponding to the mean age of the sampled population and to zero values for co-factors). The range of the 95% credibility intervals for each pixel of the spatial random effect is presented in Figure 6b. As a result of this analysis, one large hotspot of human AE disease was indicated in an area at the south-east border of Qinghai Province and at the north-west of Sichuan Province. Over a total area of 290,400 km^2^, an endemic area of 193,600 km^2^ was calculated to have a human AE prevalence higher than 1 per thousand; for human AE prevalence greater than 1% the predicted transmission zone was 67,200 km2.

**Figure 7:**
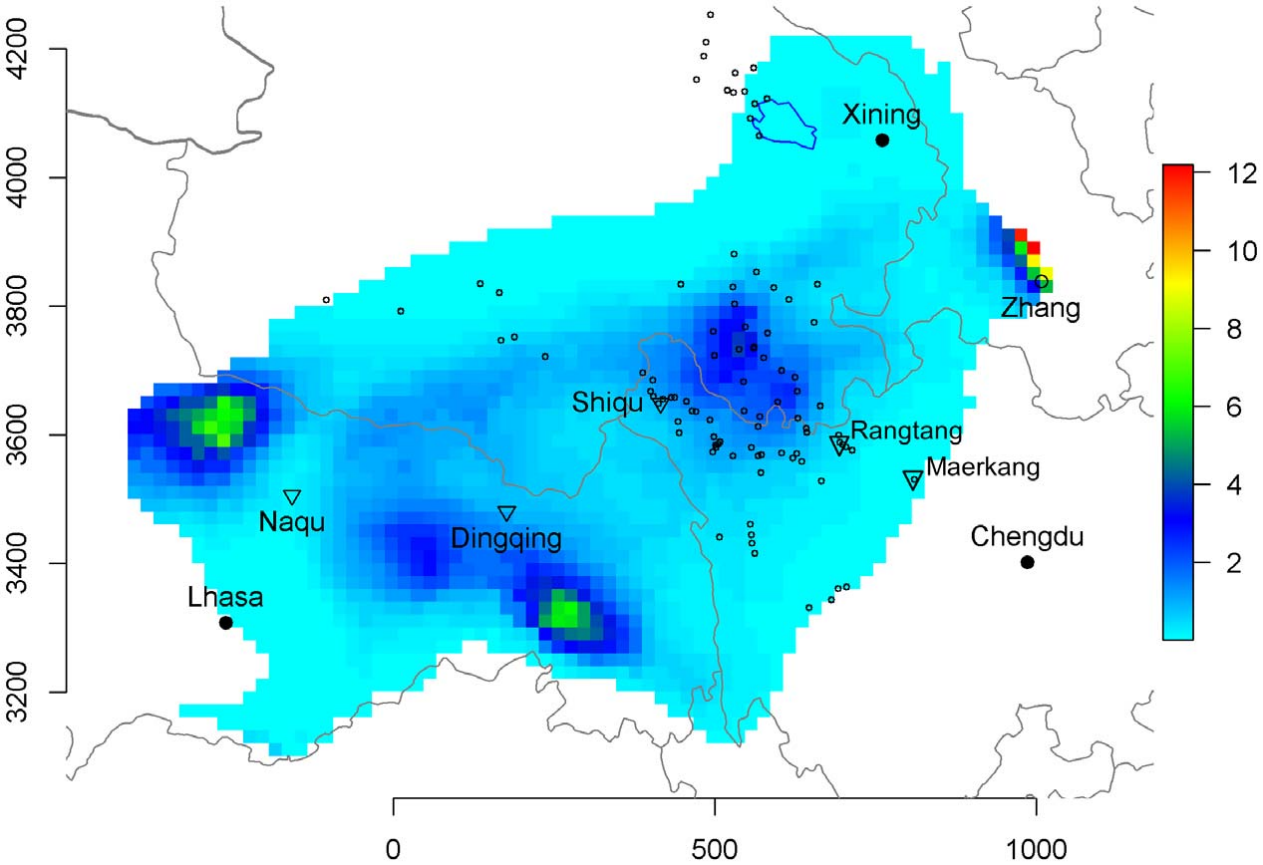
Prediction of human AE prevalence on the eastern Tibetan plateau. The vertical gauge gives the predicted prevalence in percent. Empty circles, villages surveyed in the present or earlier studies, triangles,localities cited in the text. Axes units are in kilometers (UTM tile 47).

Predictions were extrapolated across a larger area where rainfall and temperature data remained within the same ranges as the area used to train the model (Figure 7). Over a total area of 902,800 km^2^, human AE prevalence was predicted to be higher than 1 per thousand over 664,000 km^2^, and greater than 1% over 210,000 km^2^. Three other hotspots of human AE disease were predicted from the landscape model. Two were located in the Tibetan Autonomous Region (TAR), one region at the north-west of Naqu and the other to the south of Dingqing. The south Dingqing putative focus was predicted to be connected to the Qinghai-Sichuan hotspot by a crescent shape corridor of elevated prevalence. This appears to be confirmed because a recent initial screening study (n = 232 persons) in Dingqing found a very high AE prevalence of 4.7% (Feng X and Craig PS, unpublished observations). Another hotspot was predicted in Gansu at the north-east limit of the transmission area. Actually, the Gansu hotspot was the first confirmed in the late 1980s [51] (see discussion). Furthermore, the area west of Qinghai Lake was predicted as a low prevalence area, which was confirmed by the present study (this was the subset of 1975 people – 13 villages - where no AE cases could be detected, thus excluded from model fitting).

## Discussion

Zoonotic pathogens are of growing concern and the role of animal host biodiversity is both of theoretical and practical consideration regarding pathogen stability and the effect of anthropogenic perturbations on the transmission ecosystem [4], [52]. Human alveolar echinococcosis (AE) is a chronic potentially fatal hepatic infection, for which the causative pathogen *Echinococcus multilocularis*, is transmitted in indirect life-cycles involving mammalian wildlife predator-prey interactions between fox definitive hosts and small mammal intermediate hosts [6], [7]. This cestode zoonosis is known to be highly endemic in parts of Eurasia including China, and provides an excellent model to investigate the role of reservoir host diversity, landscape characters and climate for a non-vector borne zoonotic macro-parasite.

### Landscape, human AE distribution and small mammal assemblages

For continental China, we found that the spatial distribution of human AE disease was not clearly correlated to small mammal species richness, even when limiting consideration to the distribution and number of species that are known to be both potential intermediate hosts and/or agricultural/grassland pests. There was however a clear spatial overlap between the distribution of certain grassland types, and the spatial distribution of human AE. The main endemic areas corresponded to the central and eastern Tibetan meadows, those of the Tien Shan and Altai Mountains and of northern Inner Mongolia. Rainfall, altitude and temperature did not directly correlate with human AE disease distribution at continental scale.

Human AE prevalence was found to exponentially increase with the ratio of alpine meadows and decrease with the ratio of forests in our regional analysis. The resolution of small mammal atlases in China did not facilitate inclusion of biodiversity indices in regional models. However, the Qinghai-Sichuan human AE disease hotspot lies in one of the areas with the lowest regional biodiversity of small mammals in China (Figure 1). A comprehensive small mammal survey carried out in Shiqu county (see location Figure 6), in the middle of the Qinghai-Sichuan human AE hotspot, recorded 6 species only of which *Microtus* spp and *Ochotona curzoniae* were classified as pests [48]. By contrast, 15 small mammal species were found in the Rangtang/Maerkang locality of west Sichuan, in the more forested part of the study area, among which a maximum of 10 species were present in some forest habitats [53]. Some of those species potentially outbreaking were shared with the east Tibetan Shiqu area (i.e. *Ochotona cansus, Microtus irene, M. limnophilus*), but no indications of small mammal population surges were found from the survey and from local farmer interviews. Moreover human AE prevalence in these areas was lower (1.5% versus 6% in Shiqu county) [28].

### Impact of landscape on small mammal population dynamics and fox diet

Evidence of interactions between landscape and arvicolid vole population dynamics at various spatial scales has been provided in earlier studies in Western Europe. On the regional scale (area of about 2500 km^2^) larger variations in vole population densities occur where permanent grassland cover exceeded 50% and 85% of the total land for *M. arvalis* and *A. terrestris* respectively [16], [17]. In complex ecosystems, the population dynamics of small mammals is regulated by both top-down (predation, parasitism) and bottom-up (resources) forces in a multivariate context [54]–[57]. In general, this means that population surges are less likely in biodiverse communities of small mammals, in regions characterized by heterogeneous landscapes (e.g. forest mosaics). Towards the far west and north of the Tibetan plateau, the increasing aridity of Alpine steppes and semi-deserts and the more patchy distribution of Alpine meadows decrease both primary production and connectivity of optimal habitats, thus the probability of large scale small mammal outbreaks. By contrast extensive grasslands with higher grass production (Alpine ‘meadows’) of the Eastern Tibetan plateau forms optimal conditions on a regional scale for surges of potentially cyclic small mammal species such as the plateau pika (*Ochotona curzoniae*), several species of *Microtus* (*M. leucurus, M. irene, M. limnophilus*), and probably hamsters (*Cricetulus* sp.). In such contexts, definitive hosts, foxes, take advantage of the most abundant and accessible resources and specialize on them [58]–[60]. Raoul et al. [61] have shown that infection of foxes with *E. multilocularis* responds quickly and asymptotically to small increases in the densities of favored prey species. This may cause intense environmental contamination and human exposure, directly from fox feces or more likely via dogs infected from preying upon abundant (infectious) small mammal intermediate host reservoirs [62]. Such system processes may explain why, on the Tibetan plateau, regional landscape variables were found to help to predict human AE distribution.

### Co-linearities on the Tibetan plateau

The greatest difficulty in interpreting these results arises from the fact that in such regional systems, explanatory variables which may determine transmission, directly or not, are not independent. For instance, grass productivity and forest cover are correlated with rainfall and temperature, which are correlated (locally) with altitude. This may explain why apparent discrepancies were observed between univariate and multivariate statistics. For instance, most Tibetans were also livestock herders, which may have nullified the occupation variable in the multivariate analysis. Furthermore, human AE disease was found to be correlated with Alpine meadow cover in univariate statistics, but in multivariate statistics effect size of alpine meadow was smaller when the forest term was included in models.. However perfect colinearity between forest and meadow aerial covers was ruled out by examining covariation matrices (data not shown) and because discarding one of the two variables in multivariate models led to increased DICs. The spatial random effect may also have nullified other effects suggesting spatial heterogeneity in effect size or the role of other unmeasured factors (e.g. socio-economics, etc.).

### Peripheral foci, landscape and infection dynamics

Large unexplained differences between neighboring villages were observed on the Tibetan plateau. Such differences are commonly reported in most other studies in endemic AE areas, for instance in Gansu [27], [49], Ningxia [50] and France [9]. Our study shows that the Zhang county local AE focus (about 400 km^2^) in south Gansu Province lies in an area where regionally, rainfall and temperature were in the range of those found in the Qinghai-Plateau hotspot, although at a much lower altitude (2000–2800 m). The Zhang focus, first discovered in the late 1980s [51] was comprehensively described in studies carried out in the late 90s [9], [49], [63]. An overall human AE prevalence of 4.1% was reported there with some villages reaching more than 10%. Some authors [9], [27], [64] had previously provided indications that *E. multilocularis* transmission in Zhang was the consequence of a transient augmentation in shrub and grass cover in the 1980s, generated by successional growth following deforestation and triggering population outbreaks of the vole *M. limnophilus* and the hamster *C. longicaudatus*. Following 20–30 years of deforestation the parasite life-cycle is no longer being maintained in the current farmland landscape due to lack of both suitable intermediate and definitive hosts [5]. This suggest that in areas where climatic conditions are expected to be favorable, but landscape unfavorable, fine grain landscape alteration (here of anthropogenic origin) may create transitory local foci of *E. multilocularis* transmission that are prone to extinction. How the parasite may colonize such areas from larger and more stable regional foci remains open to question (mainland/island dynamics through fox movements and/or dog trade?) [5]. The present study however shows that even those risk areas may be predicted from climate and landscape analysis at medium (20 km×20 km) resolution.

In conclusion, our results indicate that the prevalence of the cestdode zoonosis, human alveolar echinococcosis, was higher in areas of relatively low small mammal biodiversity than in more diverse host communities within which they were nested. In low diversity small mammal communities, potential intermediate host species prone to population outbreaks (e.g. rodents and lagomorphs) can reach higher densities and enhance transmission. This can be a natural occurrence, as on the homogeneous meadows of the eastern Tibetan plateau where rainfall maintains relatively higher productivity than is possible on the western half of the Tibetan plateau. Conversely, it can also be the result of anthropogenic driven landscape alteration, as in south Gansu where deforestation under favorable climatic conditions led to a transitory decrease of small mammal local biodiversity, and an augmentation of habitats and landscapes favorable to one or several cyclic species of those low diversity communities. Our results support the notion that landscape, small mammal host biodiversity and their population dynamics may protect humans from *E. multilocularis* transmission by preventing population outbreaks of specific small mammal host species with subsequent consequences on prey/predator relationships and associated host/parasite transmission [65]. This mechanism is clearly different to the dilution and zooprophylaxis effects that have been described and debated elsewhere as potential utilitarian advantages of biodiversity conservation [4]

## Supporting Information

Figure S1 Altitude statistics in continental China (in a 100 km radius buffer) and human alveolar echinococcosis distribution (red lines).
(TIF)

Figure S2 Rainfall statistics (logarithm) in continental China (in a 100 km radius buffer) and human alveolar echinococcosis distribution (red lines).
(TIF)

Figure S3 Average temperature in continental China (in a 100 km radius buffer) and human alveolar echinococcosis distribution (red lines).
(TIF)

Figure S4 Land cover in continental China (ratio of total land in a 100 km radius buffer) according to GLOBAL land cover 2000 nomenclature and human alveolar echinococcosis distribution (red lines). V1, needleleaved deciduoud forest; V2, needleleaved evergreen forest; V3, broadleaved evergreen forest; V4, broadleaved deciduoud forest; V5, bush; V6, sparse woods; V7, seaside wet lands; V8, alpine and subalpine meadow; V9, slope grassland; V10, plain grassland; V11, desert grassland; V12, meadow; V13, city; V14, river; V15, lake; V16, swamp; V17, glacier; V18, bare rocks; V19, gravels; V20, desert; V21, farmland; V22, alpine and sub-alpine plain grass; V23, Mosaic of cropping; V24, Forest Mosaic/Degraded Forest;
(TIF)

Table S1 Model comparison with deviance information criterion. Spatial and random effects are depicted by spatial and pixel respectively. Tibetan, Female, Herdpeople are factors coded 0/1; ratio meadows and ratio forests are the ratio of Alpine meadows and of forest to total land within a 100 km radius buffer. P-spline term was added on age, altitude, rainfall and temperature.
(DOC)
