# Supplementary figures and images for: Drivers of Echinococcus multilocularis Transmission in China: Small Mammal Diversity, Landscape or Climate?
Source: PLoS Negl Trop Dis. 2013 Mar 7;7(3):e2045. doi: 10.1371/journal.pntd.0002045 (PMC3591347; doi:10.1371/journal.pntd.0002045)

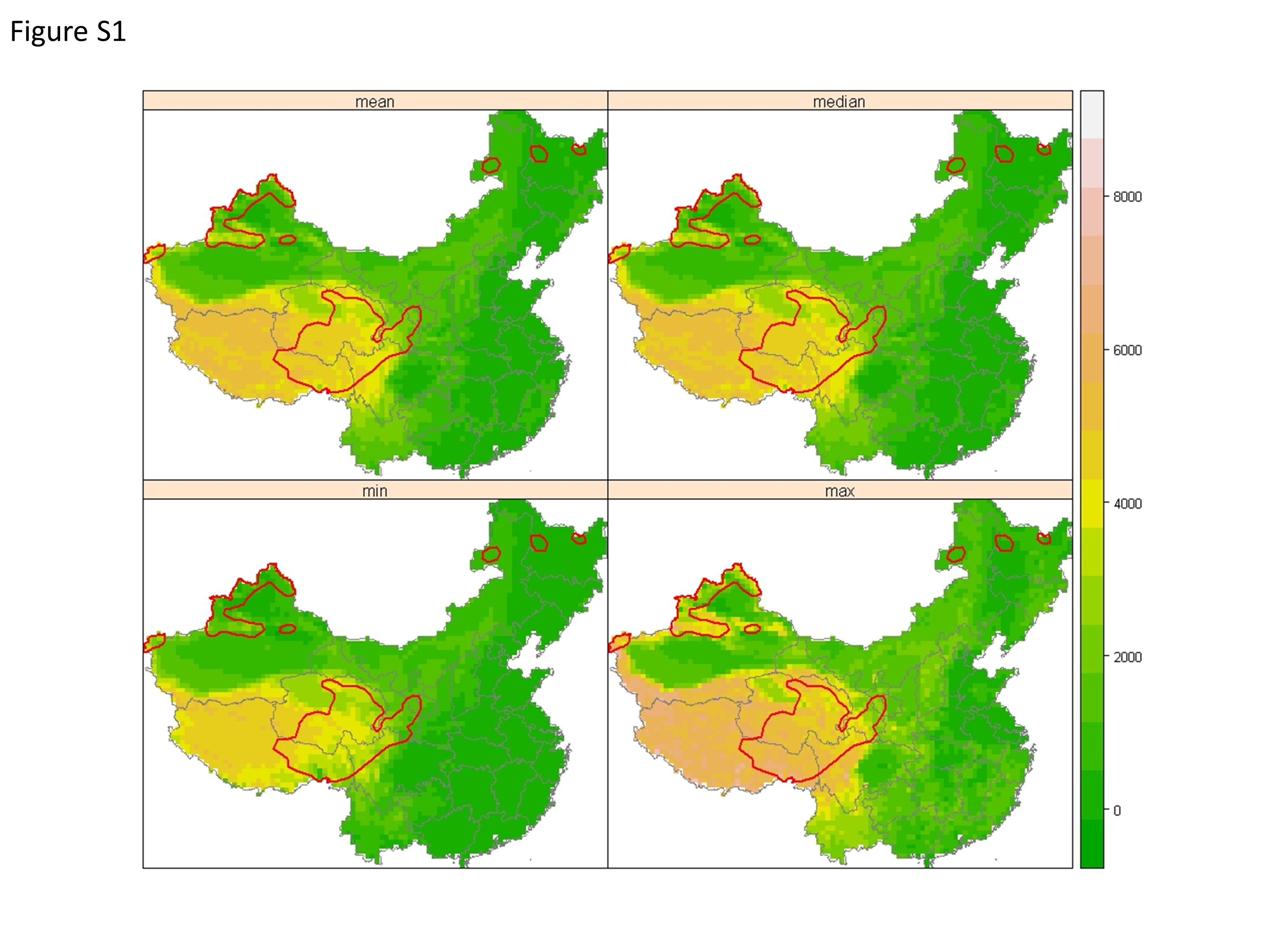

Supplement: Figure S1 — Altitude statistics in continental China (in a 100 km radius buffer) and human alveolar echinococcosis distribution (red lines). (TIF) [file pntd.0002045.s001.tif]

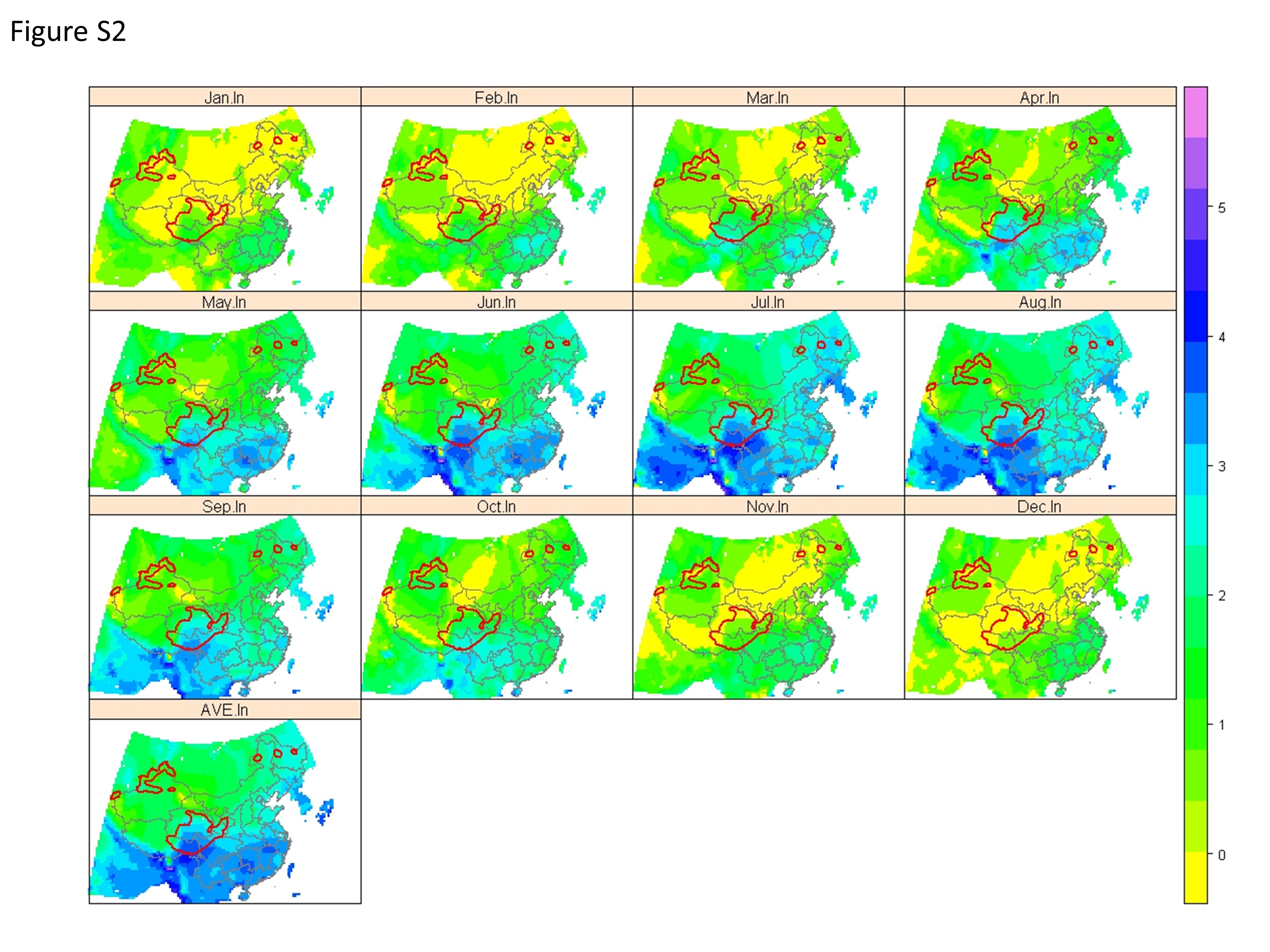

Supplement: Figure S2 — Rainfall statistics (logarithm) in continental China (in a 100 km radius buffer) and human alveolar echinococcosis distribution (red lines). (TIF) [file pntd.0002045.s002.tif]

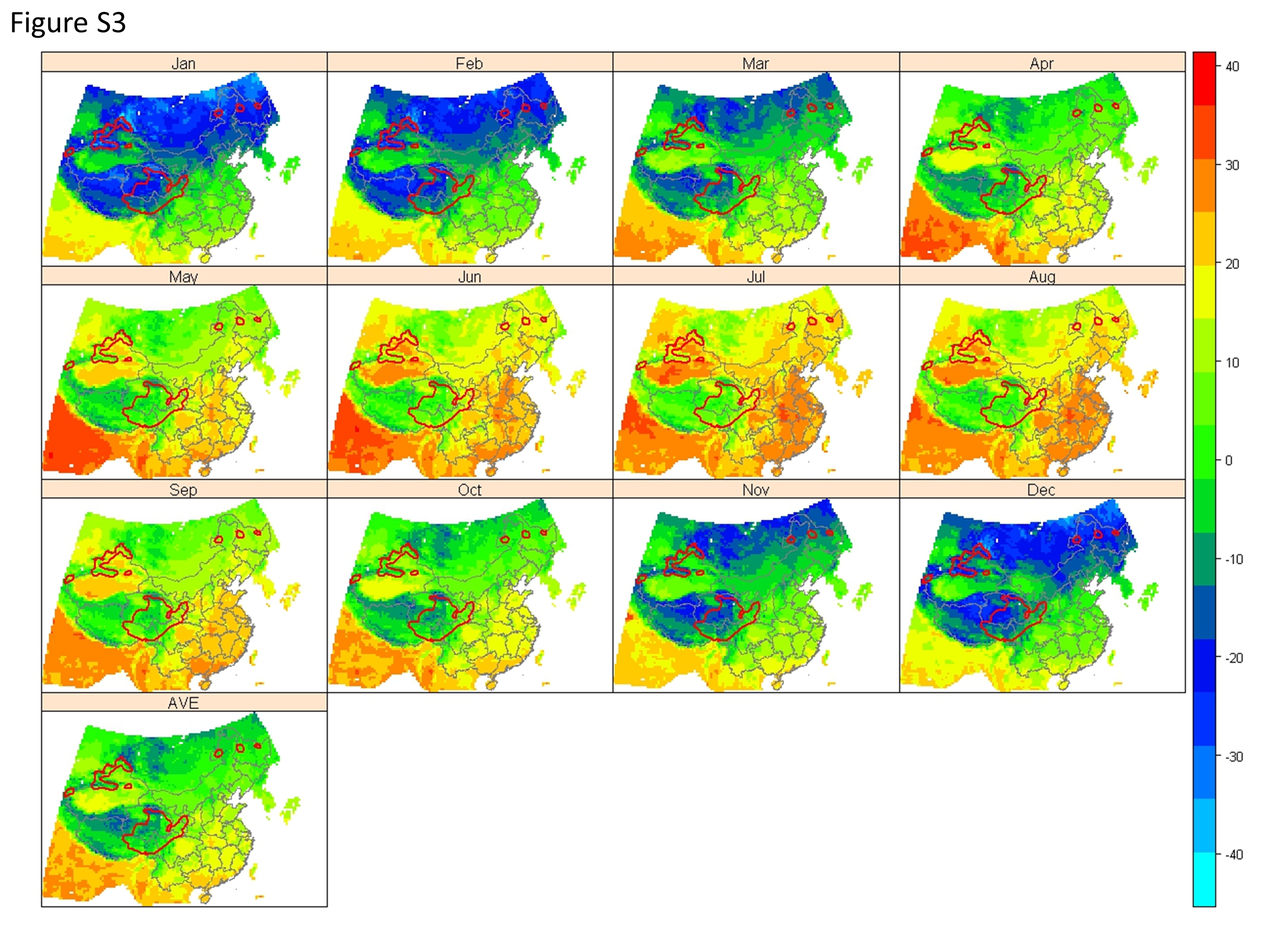

Supplement: Figure S3 — Average temperature in continental China (in a 100 km radius buffer) and human alveolar echinococcosis distribution (red lines). (TIF) [file pntd.0002045.s003.tif]

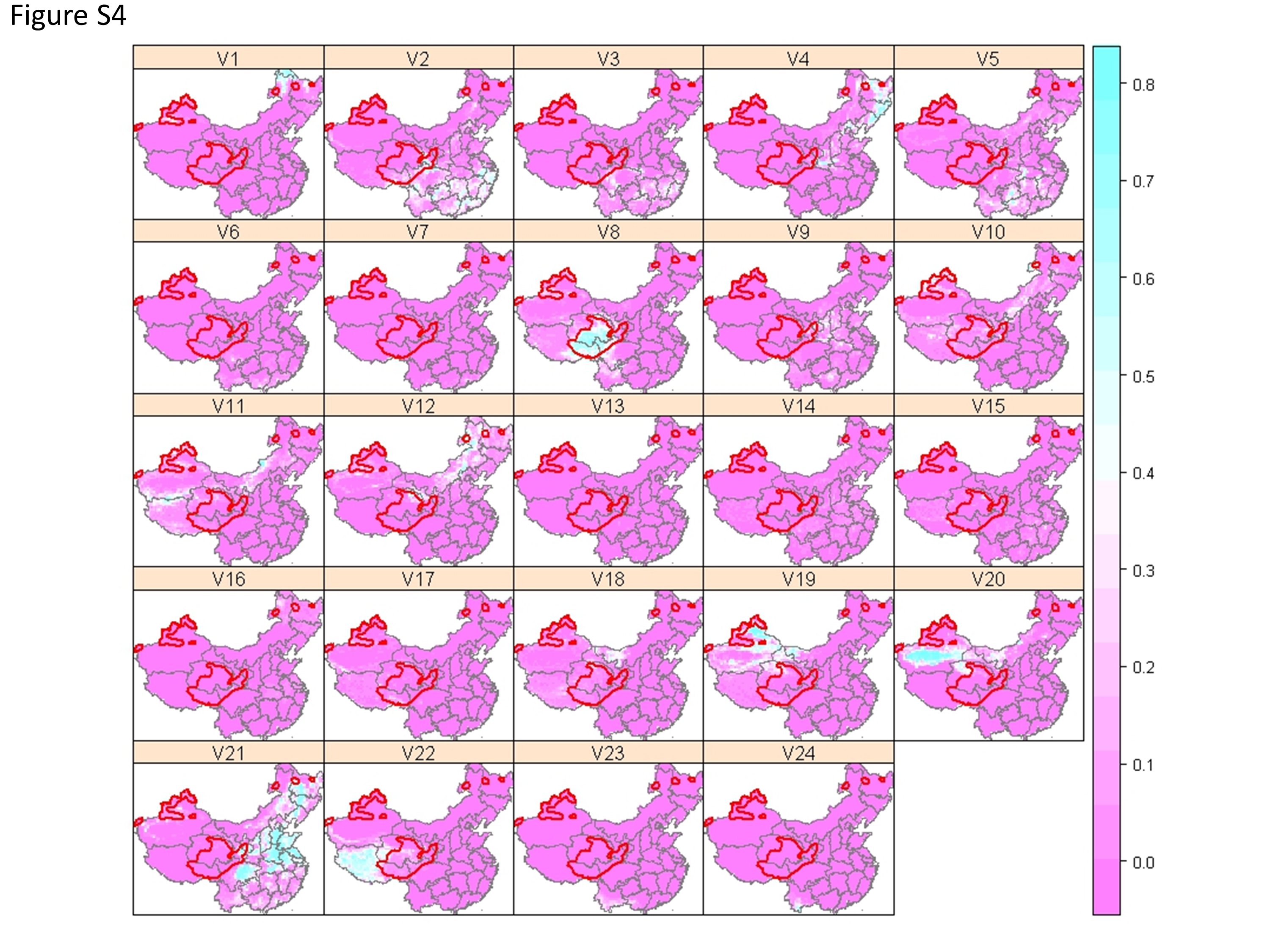

Supplement: Figure S4 — Land cover in continental China (ratio of total land in a 100 km radius buffer) according to GLOBAL land cover 2000 nomenclature and human alveolar echinococcosis distribution (red lines). V1, needleleaved deciduoud forest; V2, needleleaved evergreen forest; V3, broadleaved evergreen forest; V4, broadleaved deciduoud forest; V5, bush; V6, sparse woods; V7, seaside wet lands; V8, alpine and subalpine meadow; V9, slope grassland; V10, plain grassland; V11, desert grassland; V12, meadow; V13, city; V14, river; V15, lake; V16, swamp; V17, glacier; V18, bare rocks; V19, gravels; V20, desert; V21, farmland; V22, alpine and sub-alpine plain grass; V23, Mosaic of cropping; V24, Forest Mosaic/Degraded Forest; (TIF) [file pntd.0002045.s004.tif]
