# Supplementary material for: Drivers of Echinococcus multilocularis Transmission in China: Small Mammal Diversity, Landscape or Climate?
Source: PLoS Negl Trop Dis. 2013 Mar 7;7(3):e2045. doi: 10.1371/journal.pntd.0002045 (PMC3591347; doi:10.1371/journal.pntd.0002045)
Supplement: Table S1 — Model comparison with deviance information criterion. Spatial and random effects are depicted by spatial and pixel respectively. Tibetan, Female, Herdpeople are factors coded 0/1; ratio meadows and ratio forests are the ratio of Alpine meadows and of forest to total land within a 100 km radius buffer. P-spline term was added on age, altitude, rainfall and temperature. (DOC) [file pntd.0002045.s005.doc]

**Table S1**

| Model | tibetan | female | s(age) | herdpeople | ratio meadows | ratio forest | s(altitude) | s(rainfall) | s(temp) | spatial | pixel | DIC | deltaDIC |
| --- | --- | --- | --- | --- | --- | --- | --- | --- | --- | --- | --- | --- | --- |
| 1 | + | + | + | + | + | + |  | + |  | + |  | 4226.7 | 0.0 |
| 2 | + | + | + | + | + | + |  | + |  | + | + | 4226.8 | 0.0 |
| 3 | + | + | + | + | + | + |  | + | + | + |  | 4227.0 | 0.3 |
| 4 | + | + | + | + | + | + | + | + |  | + | + | 4227.4 | 0.6 |
| 5 | + | + | + | + | + | + |  | + | + | + | + | 4227.4 | 0.7 |
| 6 | + | + | + | + | + | + | + |  |  | + |  | 4228.9 | 2.2 |
| 7 | + | + | + | + |  | + |  |  |  | + | + | 4229.0 | 2.3 |
| 8 | + | + | + | + | + |  |  | + | + | + | + | 4229.2 | 2.5 |
| 9 | + | + | + | + |  | + |  |  |  | + |  | 4229.5 | 2.8 |
| 10 | + | + | + | + |  |  | + |  |  | + |  | 4229.5 | 2.8 |
| 11 | + | + | + | + | + | + |  |  |  | + | + | 4229.6 | 2.9 |
| 12 | + | + | + | + | + | + |  |  |  | + |  | 4229.7 | 3.0 |
| 13 | + | + | + | + |  | + |  | + |  | + | + | 4229.8 | 3.1 |
| 14 | + | + | + | + |  |  |  | + | + | + | + | 4229.9 | 3.2 |
| 15 | + | + | + | + |  | + | + |  |  | + | + | 4229.9 | 3.2 |
| 16 | + | + | + | + |  | + |  | + | + | + | + | 4230.2 | 3.5 |
| 17 | + | + | + | + |  |  | + |  |  | + | + | 4230.4 | 3.6 |
| 18 | + | + | + | + | + | + | + |  |  | + | + | 4230.4 | 3.7 |
| 19 | + | + | + | + |  |  |  |  |  | + | + | 4230.5 | 3.8 |
| 20 | + | + | + | + | + |  |  |  |  | + |  | 4230.8 | 4.1 |
| 21 | + | + | + | + | + |  |  |  |  | + | + | 4230.9 | 4.2 |
| 22 | + | + | + | + | + |  | + |  |  | + | + | 4231.0 | 4.3 |
| 23 | + | + | + | + | + | + |  | + | + |  | + | 4231.6 | 4.9 |
| 24 | + | + | + | + |  | + | + |  |  | + |  | 4235.8 | 9.1 |
| 25 | + | + | + |  |  | + |  |  |  | + | + | 4235.9 | 9.2 |
| 26 | + | + | + |  | + | + |  |  |  | + | + | 4236.1 | 9.4 |
| 27 | + | + | + |  | + |  |  |  |  | + | + | 4236.5 | 9.8 |
| 28 | + | + | + |  |  |  |  |  |  | + | + | 4236.8 | 10.1 |
| 29 | + | + | + |  | + |  |  |  |  | + |  | 4237.3 | 10.5 |
| 30 | + | + | + | + | + |  |  |  |  |  | + | 4244.8 | 18.1 |
| 31 | + | + | + |  | + |  |  |  |  |  | + | 4250.0 | 23.3 |
| 32 | + | + | + | + |  | + |  |  |  |  |  | 4600.7 | 374.0 |
| 33 | + | + | + |  | + |  |  |  |  |  |  | 4621.4 | 394.7 |
